# Exergaming in a Moving Virtual World to Train Vestibular Functions and Gait; a Proof-of-Concept-Study With Older Adults

**DOI:** 10.3389/fphys.2018.00988

**Published:** 2018-07-31

**Authors:** Jaap Swanenburg, Karin Wild, Dominik Straumann, Eling D. de Bruin

**Affiliations:** ^1^Physiotherapy and Occupational Therapy Research Center, Directorate of Research and Education, University Hospital Zurich, University of Zurich, Zurich, Switzerland; ^2^Integrative Spinal Research (ISR), Department of Chiropractic Medicine, Balgrist University Hospital, Zurich, Switzerland; ^3^Department Health Sciences and Technology, Institute of Human Movement Sciences and Sport, ETH Zurich, Zurich, Switzerland; ^4^Department of Neurology, University Hospital Zurich, University of Zurich, Zurich, Switzerland; ^5^Division of Physiotherapy, Department of Neurobiology, Care Sciences and Society, Karolinska Institutet, Stockholm, Sweden

**Keywords:** vestibular loss, exergaming, older adult, head turns, dynamic visual acuity

## Abstract

**Background:** The use of Exergames designed to improve physical and cognitive functioning is relatively new in rehabilitation. Exergaming allows the training of skills, the handling of tools, and procedures; however, often, the potential of these aspects are not assessed before they are adopted in clinical settings. This study aimed at exploring the effects of exergaming on vestibular functions and gait in healthy community dwelling older adults using a proof-of-concept study design registered under ClinicalTrials.gov NCT03160352.

**Methods:** A pre-test-post-test one-group study design comprising 10 older adults (mean age of 73.5 ± 7.6 years, four males) investigated the feasibility of eight exergaming training sessions (for 160 min) and the effects on dynamic visual acuity (DVA), functional gait assessment (FGA), and extended timed get-up-and-go (ETGUG). The simulator sickness questionnaire (SSQ) and the game scores were evaluated for the feasibility of the intervention. Wilcoxon test and Cohen’s d (d) were chosen to test for differences and for effect size estimation.

**Results:** Exergaming led to a significantly improved DVA (*z* = −2.50, *p* = 0.01, *d* = 1.35) with improvements in 9 out of 10 participants. In addition, the FGA significantly improved with a large effect size (*z* = −2.25, *p* = 0.02, *d* = 1.17). Specifically, component tasks such as walking with horizontal head turns (*p* = 0.03), gait with a narrow base of support (*p* = 0.03), ambulating backward (*p* = 0.05) significantly improved. The ETGUG component task Gait initiation significantly improved (*p* = 0.04). No change was found in gait speed and SSQ. The game scores of the participants improved continuously during the course of the intervention for every game.

**Discussion:** This proof-of-concept study suggests that the use of exergaming that requires active stepping movements and that contains moving game projection is feasible and facilitates gaze stability during head movements in healthy community dwelling older adults. Aspects of functional gait and gait initiation also improved. Future research aimed at testing this exergaming intervention in patients suffering from vestibular impairments is warranted.

## Introduction

A decline in vestibular function with increasing age is observable in the general population (Agrawal et al., 2009; Tjernstrom et al., 2016). The prevalence of vestibular dysfunction is about 35% for those 40 years of age and above and reaches 85% in individuals aged 80 and above (Agrawal et al., 2013; Arshad and Seemungal, 2016). This decline may lead to vertigo, dizziness, and poor postural stability (Swanenburg et al., 2009; Deveze et al., 2014). Vestibular disorders can cause oscillopsia during head movements, decreased dynamic visual acuity (DVA), and reduced postural stability especially during locomotion (Herdman et al., 2003; Lacour and Bernard-Demanze, 2015). Chronic peripheral vestibular hypofunction may lead to a change in gaze behavior (Swanenburg et al., 2017a).

In general, vestibular rehabilitation and physical activity improve the symptoms related to vestibular disorders (Agrawal et al., 2013; Bergeron et al., 2015; Muinos and Ballesteros, 2015). Vestibular rehabilitation is focused on the facilitation of the maximal use of any remaining vestibular function, improvement of gaze and postural stability by the use of visual and somatosensory cues, and improvement of home and workplace safety (Calder and Jacobson, 2000). One additional important goal of vestibular rehabilitation is the improvement of gait, especially during head movements (Han et al., 2011) as a measure to prevent falls in individuals with vestibular dysfunction (Agrawal et al., 2013). To minimize the sensation of dizziness, patients tend to turn the trunk or head as little as possible (Whitney et al., 2016), which may affect postural balance and walking on stairs (Cromwell and Wellmon, 2001; Swanenburg et al., 2017b).

Computer technology that simulates real-life learning and that allows for the increased intensity of training while providing augmented three-dimensional and direct sensorial feedback is coined Virtual Reality (VR) (Saposnik et al., 2010a,b). This is a type of technology which allows users to interact with a computer-generated scenario, a.k.a. “a virtual world,” and to make corrections while performing the exercise task (Saposnik et al., 2010a,b). A 2007 Cochrane review pointed out that patients with mobility problems may transfer the training effects obtained in a virtual environment to real-life (Erren-Wolters et al., 2007), an approach also believed to be beneficial for vestibular rehabilitation (Deveze et al., 2014). The use of Exergames, technology-driven games that oblige players to be physically active to play the game (Witherspoon, 2013), has the additional advantage of providing a motivational and a pleasant way of training for the patients (Bergeron et al., 2015; Meldrum et al., 2015).

This study aimed at exploring the effects of exergaming on vestibular functions and gait in healthy community dwelling older adults using a proof-of-concept study design (Justice et al., 2016). We performed a trial to determine whether a purpose developed VR-based exergame intervention has sufficient dose level to induce a response (Thabane et al., 2010) in older adults. For this purpose conventional key exercises (Han et al., 2011; Deveze et al., 2014) were transposed to captivating Exergames. This study precedes a future clinical trial with identified vestibular patients and aims to test the effectiveness of the Exergame rehabilitation approach.

## Materials and Methods

### Participants

For the present pilot study, 10 healthy-by-self-report older adults with an expected age-related degeneration of the vestibular system were recruited from the community. Measurements were obtained at the Department of Neurology, University Hospital Zurich, Switzerland. The exergame sessions were executed at ETH Hönggerberg, Zurich, Switzerland. All the participants were above 65 years of age. The exclusion criteria were (1) walking disability (independent walking ability <10 meters), (2) acute pain, (3) uncontrollable cardiovascular disease (e.g., high blood pressure), and (4) weakness resulting from known neurological problems. A written informed consent was obtained from all the participants. The ethics committee of Canton Zurich approved the study (BASEC 2016–1220), and the study is registered under ClinicalTrials.gov (NCT03160352).

### Intervention

**FIGURE 1:**
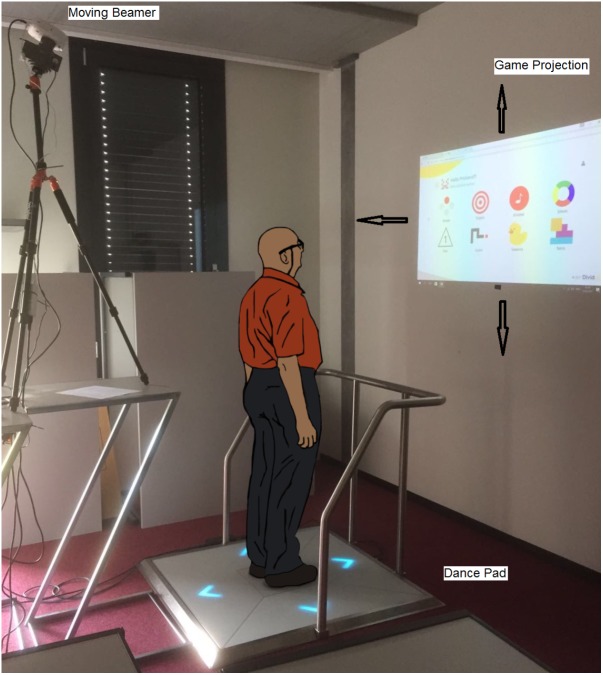
The Senso exercise system with moving beamer (arrows show motion extent of game projection).

Based on existing exercise recommendations (Han et al., 2011; Deveze et al., 2014), the exergame intervention developed included challenging conditions such as head turns and unsupported locomotion, and targeted the recovery of visual dynamic acuity and gait. The exergames used are useful for improving divided attention, working memory, inhibition, shifting, and physical functioning. The participants were expected to play the games in a standing position without any support. Dynamic changing of the projection surface while playing the exergame was purposefully integrated in the program (the projection could move up, down, to the left, or right) as a measure to ensure the progression of exercise difficulty. This encouraged the participants to turn their head to keep watching the projection while at the same time maintaining their body position to successfully play the game (**Figure 1**). Head-trunk turns were exercised by having the participant stand at a 90° angle relative to a wall, on which the game was projected. Thus, subjects had to turn their heads in the direction of the projection. **Table 1** details the exercise protocol.

**Table 1:** The exercise protocol.

Game	Exercise sessions							
	1	2	3	4	5	6	7	8
Simple A	I	I	I	I	I	I	I	I
Simple B	0	0	0	0	0	0	0	0
Target A	I	I	II	II	IV	V	IV	V
Target B	II	II	IV	V	V	IV	V	IV
Flexi A	I	I	II	II	II	II	IV	V
Flexi B	II	II	III	III	IV	V	V	IV
Snake A	I	I	I	I	I	I	I	I
Snake B	II	II	II	II	II	II	II	II

*0 = no projection, only looking down at the plate. I = static projection, looking forward. II = dynamic horizontal projection, rotating head horizontally. III = dynamic horizontal + vertical projection, rotating head horizontally + vertically. IV = right – participant rotated by 90° to the right, head rotated to the left. V = left – participant rotated by 90° to the left, head rotated to the right.*

### Intervention Design

Eight exergame sessions had to be completed, preceded by a baseline and followed by a post-intervention measurements session that followed directly after the eighth training. Each of the eight exercise sessions lasted 40 min, with an actual “time-on-task” exercise duration of 20 min. This resulted in a cumulative total intervention exercise time of 160 min. This duration was chosen based on a recommended minimum of 150 min for positive outcomes of VR interventions in vestibular disorders (Bergeron et al., 2015). During their scheduled individual exergame sessions, the participants received one-on-one attention from the research assistant.

To maximize adherence, the participants, together with the research assistant, set up and planned their individualized program over a 4-week period. As documented in **Table 1**, the training principles of progression and overload (Klatt et al., 2015; Schattin et al., 2016) were explicitly considered and managed by the research assistant. One intervention session included four games, with each game being played twice. One exergame round lasted 150 s. The participants trained on a Senso exergame system (dividat, Schindellegi, Switzerland; **Figure 1**). The participants trigger sensors positioned on a base plate through body movements and, by doing that, apply targeted forces as well as steps of which the dynamics are recorded. The sensor plate was connected to a desktop computer and beamer by USB. Electronic sensors in the dance pad detected position and timing information that provided the participants with real-time visual and auditory feedback on exergame performance. Content wise, the Senso exergames are designed to train divided attention, working memory, inhibition, attention shifting, and postural control. A moveable beamer (LG PW800G) was used to project the exergames on a white wall. The visual projection angles measured ± 81° in width and ± 54° in height (**Figure 1**). To promote head movement while exercising, the projection direction of the beamer could be turned ± 45° horizontally and tilted vertically ± 15° by using a remote-controlled Tilt Power Panner (Maxwell MPR–202). The horizontal tilting speed was 6°per second and vertically it was 2°per second. The research assistant controlled the patient’s head movement visually.

### Exergames

#### Simple

**FIGURE 2:**
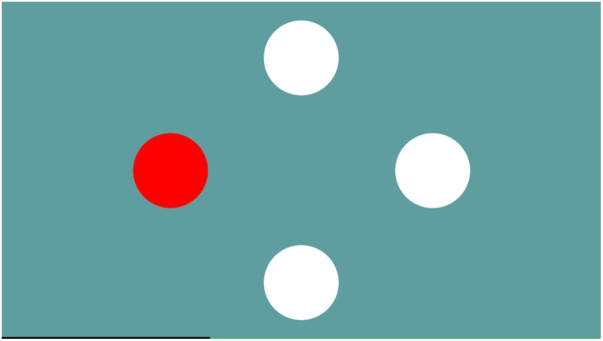
The game Simple trains focussed attention – the ability to concentrate on certain stimuli and react as quickly as possible to them.

This exergame was used as a warm-up and to teach how to deal with the SENSO plate. The game screen showed four circles representing four arrows on the plate. As soon as one circle turned red in random order, the participant had to press the corresponding arrow as quickly as possible. The game progressively adapted the difficulty by speeding up (**Figure 2**).

#### Targets

**FIGURE 3:**
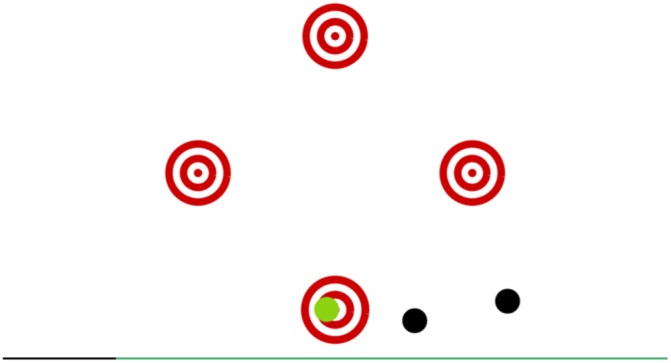
The game Targets which helps training reaction time (speed and accuracy).

The game screen showed four red circled targets representing four arrows on the plate. During the game, black balls were flying through the screen and were coming from random directions. The task was to press the corresponding arrow as soon as a ball blotted out a central target. More points were credited whenever the ball was closer to the center of the target circle. In the case of maximum precision, an auditory feedback was given. The game progressively adapted the difficulty by speeding up (**Figure 3**).

#### Flexi

**FIGURE 4:**
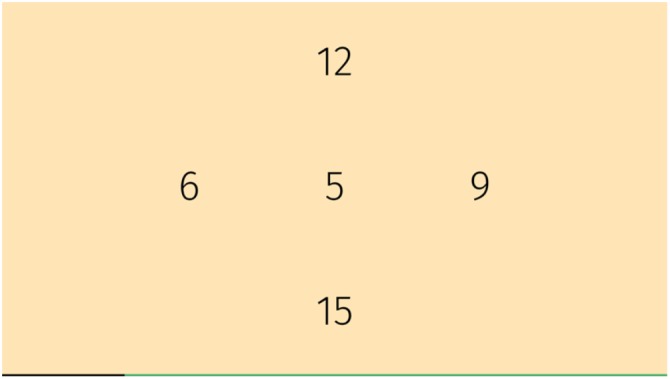
Flexi supports training of shifting attention.

The game screen showed four numbers representing four arrows on the plate (**Figure 4**). An additional number was projected in the center. This game consisted of two parts. In the first part, the participant had to play that number which was identical to the center one. In the second part, the center number was surrounded by a circle or a triangle and the participant had to find the same number as shown in the center but with the opposite symbol circle or triangle. The game progressively adapted to difficulty by speeding up.

#### Snake

**FIGURE 5:**
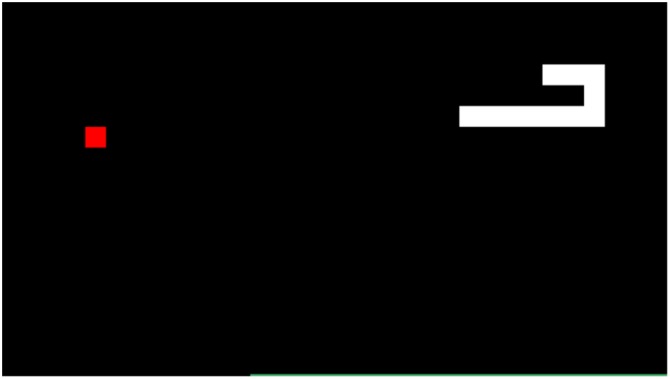
The Snake game which supports training of spatial orientation in a 2D virtual environment.

Every training session was completed with the snake game. In the game “Snake,” the participant played with the snake’s perspective, which was represented by a white moving line (**Figure 5**). The task was to direct the snake to the red square by touching the corresponding arrows. With each successful hit, the snake progressively increased in length. When the snake touched one edge of the screen, it appeared and continued moving on the opposite side of the screen with the same speed and direction.

### Measurements

#### Dynamic Visual Acuity (DVA)

Dynamic visual acuity is the measurement of visual acuity during head movement relative to baseline static visual acuity (SVA) (Herdman et al., 1998; Schubert et al., 2006). The test gives information on the vestibulo-ocular reflex (VOR) performance and semicircular canal function (Vital et al., 2010). For horizontal testing, the head rotation was assessed in both directions (Petersen et al., 2013). DVA assessment was performed using the standard optotype “Landolt ring” as a visual target with eight different orientations. The DVA testing system consisted of a personal computer with a 19-inch monitor (1280 × 1024 pixels, 75 Hz) and a Sparkfun velocity sensor (Sparkfun Electronics, Boulder, CO, United States), which was fixed on a headset to the participant’s head (Vital et al., 2010; Wettstein et al., 2016). The monitor was placed at a distance of 5 meters in front of the participant. By subtracting SVA from DVA, the VA-Loss was calculated; a measure of the decrement of visual acuity during motion (Vital et al., 2010). Larger VA-Loss values indicate dysfunction (Wettstein et al., 2016). Minimum acceptable head testing velocity was 150°/s. Since all the participants were healthy-by-self-report community dwellers the DVA results of the left and the right side were combined.

#### Functional Gait Assessment (FGA)

Functional gait assessment is used to measure disturbances in balance and gait (Wrisley et al., 2004) and includes the following 10 items (Walker et al., 2007): (1) Gait level surface, (2) Change in gait speed, (3) Gait with horizontal head turns, (4) Gait with vertical head turns, (5) Gait and pivot turn, (6) Step over obstacle, (7) Gait with narrow base of support, (8) Gait with eyes closed, (9) Ambulating backward, and (10) Steps. Each item was scored on a four-point ordinal scale with scores of 0, 1, 2, and 3 (Maximum total score = 30). Higher scores represent better balance and gait capacity (Wrisley et al., 2004).

#### Extended Timed Get-Up-and-Go (ETGUG)

The extended timed get-up-and-go (ETGUG) test measures the time needed to complete a series of functionally important tasks with a multimemory stopwatch: (1) Rising from a chair, (2) Initiating gait, (3) Walking 6 m straight, (4) Turning around, (5) Walking 6 m back toward the chair, and (6) Sitting down. The time for the individual tasks and the overall time reflect functional mobility (Swanenburg et al., 2014). The mean gait speed of the participants was derived from tasks 3 (walk 1) and 5 (walk 2) by adding the time needed for these two component tasks and dividing by 12 (Swanenburg et al., 2014).

#### Simulator Sickness Questionnaire (SSQ)

The simulator sickness questionnaire (SSQ) questionnaire assesses cyber or VR sickness (Keshavarz and Hecht, 2011). It contains 16 items which had to be weighed by the participants in terms of severity on a four-level scale with the options including “none,” “slight,” “moderate,” and “severe” (Kennedy et al., 1993) (Minimum score = 0, Maximum total score = 48).

The participants completed the SSQ immediately after each exercise session, and during the evening hours of the same exercise day for assessing possible delayed symptoms.

#### Game Scores

The game scores and attendance of the exercise sessions were recorded in training logs. In addition, hits and misses were calculated for the game “Targets.”

### Statistical Analysis

Descriptive statistics were used to describe the demographic characteristics of the sample. The Shapiro–Wilk test was used to test the normality of data distribution. In case of non-normal distribution, the Wilcoxon test was chosen to test for pre- to post-intervention differences in the selected outcomes. Additionally, for the effect size Cohen’s d (d) was calculated. Cohen’s d is interpreted as d: 0.1–0.3: “small effect”; >0.3–0.5: “medium effect”; >0.5: “large effect.” IBM SPSS Statistics 25 for Windows (Inc., Chicago, IL, United States) was used for all the statistical analyses.

## Results

Ten (mean age 73.5 ± 7.6 years; mean weight 73.7 ± 18.3 kg; mean height 172.3 ± 9.4 cm, four males) healthy-by-self-report community dwellers were analyzed. No participant dropped out during the exergame intervention period. Adherence to the program was 98%. Because of time management conflicts, one participant was not able to complete the last two out of scheduled eight training sessions. Missing exergame scores for this individual were replaced by the mean value of all the other participants. In one participant, the results of the DVA post-measurements on the right showed an artifact; hence the result of post-measurement on the left side was used for the calculations.

**FIGURE 6:**
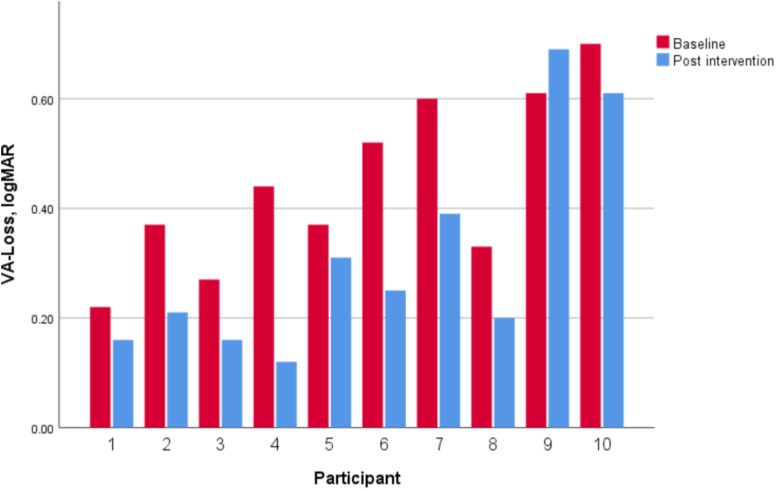
Baseline and post-intervention VA-Loss results of each participant.

Dynamic visual acuity showed a significant decrease from 0.44 ± 0.16 logMAR at baseline to 0.31 ± 0.20 logMAR post-intervention and a large concomitant effect size (*z* = −2.50, *p* = 0.01, *d* = 1.35). Nine of the 10 participants showed an improvement (**Figure 6**).

The FGA total score showed a significant improvement and a large effect size (*z* = −2.25, *p* = 0.02, *d* = 1.17). The tasks Gait with horizontal head turns (*p* = 0.03), Gait with a narrow base of support (*p* = 0.03) and Ambulating backward (*p* = 0.05) significantly improved. **Table 2** shows the results of all the FGA tasks.

**Table 2:** The results of FGA all tasks and total score.

	Baseline Mean	Post-intervention Mean	*p*	*d*
Gait on level surface; (SD) Range	3.0 (0.0) 3/3	3.0 (0.0) 3/3	1.00	0
Change in gait speed; (SD) Range	2.9 (0.3) 2/3	3.0 (0.0) 3/3	0.32	0.46
Gait with horizontal head turns (SD) Range	2.1 (0.6) 1/3	2.7 (0.5) 2/3	0.03^∗^	1.08
Gait with vertical head turns (SD) Range	2.7 (0.5) 2/3	2.8 (0.4) 2/3	0.56	0.26
Gait with pivot turn (SD) Range	2.6 (0.5) 2/3	2.9 (0.3) 2/3	0.08	0.84
Step over obstacle (SD) Range	2.8 (0.4) 2/3	3.0 (0.0) 3/3	0.16	0.67
Gait with narrow base of support (SD) Range	2.3 (0.7) 1/3	2.8 (0.4) 2/3	0.03^∗^	1.16
Gait with eyes closed (SD) Range	2.7 (0.5) 2/3	2.6 (0.5) 2/3	0.57	0.26
Ambulating backward (SD) Range	2.6 (0.5) 2/3	3.0 (0.0) 3/3	0.05^∗^	1.00
Steps (SD) Range	2.7 (0.5) 2/3	2.6 (0.5) 2/3	0.56	0.26
Total (SD) Range	26.4 (3.0) 21/30	28.4 (1.6) 26/30	0.02^∗^	1.167

*SD = standard deviation; Range = min/max; p = Wilcoxen Signed Rank Test; ^∗^significant difference (p < 0.05); d = Cohen’s d.*

The total ETGUG score showed no change and a medium effect size (*z* = −0.87, *p* = 0.39, *d* = 0.25). The second component task “initiating gait” showed a significant reduction in the time required with a large effect size (*p* = 0.04, *d* = 1.06). **Table 3** summarizes the results of all the ETGUG component tasks.

**Table 3:** Results of the ETGUG all tasks and total score.

	Baseline Mean	Post-intervention Mean	*p*	*d*
Sit-to-stand; sec (SD) Range	1.0 (0.3) 0.7/1.6	1.0 (0.2) 0.7/1.3	0.76	0.14
Gait initiation; sec (SD) Range	1.8 (0.5) 1.1/2.5	1.4 (0.5) 1.0/2.5	0.04^∗^	1.06
Walk 1; sec (SD) Range	4.4 (0.8) 3.6/6.4	4.1 (0.7) 3.4/5.8	0.24	0.54
Turn around; sec (SD) Range	4.2 (0.6) 3.6/5.3	4.2 (0.7) 3.6/5.7	0.72	0.16
Walk 2; sec (SD) Range	4.2 (0.7) 3.2/5.7	4.0 (0.6) 3.4/4.9	0.51	0.30
Slow down, stop, turnaround, and sit down; sec (SD) Range	3.3 (0.4) 2.8/3.9	3.2 (0.5) 2.9/4.4	0.80	0.11
Total time; sec (SD) Range, min/max	18.9 (2.6) 16.2/24.7	17.9 (2.7) 16.3/23.6	0.39	0.40
Mean gait speed; m/s (SD) Range, min/max	1.4 (0.2) 1.0/1.8	1.5 (0.2) 1.1/1.9	0.58	0.25

*SD = standard deviation; Range = min/max; sec = seconds; p = Wilcoxen Signed Rank Test; ^∗^significant difference (p < 0.05); d = Cohen’s d.*

All the participants scored low on the SSQ immediately after each intervention session. They mainly recorded that they began sweating during the exercise. During the evening hours of the same day, none of the participants experienced any symptom. **Table 4** presents the results of all the SSQ measurements.

**Table 4:** Mean Scores of the Simulator Sickness Questionnaire after each exercise session.

	Exercise sessions							
	1	2	3	4	5	6	7	8
Mean	3.4	1.7	1.7	2.0	2.0	1.8	1.9	1.5
(SD)	(3.6)	(1.8)	(1.8)	(2.1)	(1.7)	(1.5)	(1.9)	(1.6)
Range	0/11	0/5	0/5	0/5	0/5	0/4	0/5	0/4

The participant’s game scores showed continuous improvement in game performance over the course of the intervention period in every game. The **Supplementary File** reports the scores of all the games and all the sessions. Data underlying the analysis presented in the manuscript can be found in **Supplementary Table S1**.

## Discussion

This proof-of-concept study investigated a new VR-based exergame mode using captivating exergames with the aim of testing the effectiveness of the rehabilitation approach in older adults. The results show that the use of exergaming facilitates gaze stability during head movement as measured by the computerized DVA test. Exergaming with promoted head turns and unsupported locomotion resulted in an improvement in nine out of 10 participants. This indicates that the purpose developed VR-based exergame intervention had sufficient dose level to induce a response. Before the intervention, our group of older adults was with DVA values of 0.44 ± 0.16 above the expected value for their age group compared with the in-house reference values and published normative data (Li et al., 2014; Riska and Hall, 2016). The DVA values of five participants changed to values below their age group DVA reference value (Riska and Hall, 2016) following the 4-weeks exergame period. Though this change was within the limits of minimal detectable change (Riska and Hall, 2016), in combination with the observed effect size and considering that the trainees were healthy-by-self-report, this indicates that the exergaming exercises had a positive effect on age-related DVA. Furthermore, this finding is in line with a similar effect of the slowing down of rapid DVA age-related decline found previously in physically active elderly individuals (Long and Crambert, 1990). In a wider context, older athletes practicing martial arts were shown to have better DVA than non-athletes did, which led to the conclusion of the importance of a physically active lifestyle with regular exercise when age-related perceptual decline should be counteracted (Muinos and Ballesteros, 2015). In sum, our findings in combination with scientific reports warrant performance of an exergame intervention in individuals with diagnosed vestibular dysfunction.

The selection of participants, without the typical symptoms of vestibular loss observable in patients, enabled the testing of the effects of the exergame program on normal vestibular functions. The use of exergaming as a therapeutic means supports improved exercise adaptation to the individual patient level and allows high controllable repetitions of exercises in vestibular rehabilitation. In addition, the exergaming setup of the present study promotes the idea of executing locomotion and head movements at the same time in a safe environment in these participants. In a future clinical trial, individuals with uni- and or bilateral vestibular hypofunction should be included. The result of our study, together with normative data previously reported (Li et al., 2014), allow the basic step (Gupta et al., 2016) of calculating the amount of patients needed to be included in such a clinical trial. To avoid a type I or II error, and based on the values reported in the literature for the National Institutes of Health Toolbox computerized test of DVA (Li et al., 2014), an estimated total sample size of 26 participants (13 per group) for a two group pretest–posttest design would be needed. This would result in 80% power at α-level 0.05. Furthermore, this is based on the assumption that the standard deviation of the response variable is 0.222. To account for attrition over time, the required sample size should increase somewhat. However, we have to stress that this sample size calculation has to be cautiously interpreted because our estimates may be biased because of the limited sample size (Thabane et al., 2010) of patients that were compared against the normative values (Li et al., 2014).

The DVA improvements observed in the present study link well with the improvements found in the FGA and the ETGUG. The elderly individuals in this study showed a significant improvement in FGA performance (Walker et al., 2007). The total score and the tasks “horizontal head turns,” “walking narrow base,” and “ambulating backward” improved significantly. The tasks that improved can be associated with the moves the participants had to make during the exergaming. The task “turning head during walking” can be associated with the provoked head turns during the exergaming. The task “walking narrow base” can be connected to the starting and returning to the feet into narrow base after pushing the pressure-sensitive plate in front. The “ambulating backward” can be linked to the backward pushing tasks on the pressure-sensitive plate during the exergaming. In addition, with increasing age, there seems to be a reduction in stable gait initiation, which leads to an increase in the risk of falling during gait initiation (Polcyn et al., 1998). Other than the improvements seen in the FGA, the participants also showed a significant improvement in gait initiation confirming previously observed improvements in older adults with this type of intervention (Pichierri et al., 2012). Gait initiation is a highly challenging task requiring a combination of several sensory information sources; e.g., from the vestibular, somatosensory, and visual systems (Yiou et al., 2017), and has been identified as affected in people with vestibular impairments (Sasaki et al., 2001; Henriksson et al., 2011). It seems that exergaming as performed in the present study facilitates the initiation of gait. A meta-analysis showed that exergaming improves executive functions, attentional processing, and visuospatial skills (Stanmore et al., 2017). It can be hypothesized that through the use of exergaming where the gamers have to be physically active to play the game with their feet, acquired skills lead to transfer effects in the form of improved gait initiation.

The successful use of exergaming in vestibular rehabilitation has been implemented previously (Bergeron et al., 2015; Micarelli et al., 2017). Micarelli et al. (2017) found a non-significant improvement in the VOR gain in patients with unilateral vestibular hypofunction by using a self-assessed gaming procedure with a head-mounted device (Micarelli et al., 2017). The patients with Ménière’s disease in this study showed a decreasing trend in their center of gravity sway after three-dimensional VR vestibular rehabilitation (Hsu et al., 2017). In the study by Micarelli et al. (2017) active head movements were executed in a safe sitting position (Micarelli et al., 2017). This study also promoted active head movements, however, in unsupported locomotion while standing, which pictures a more ecologically valid daily living situation.

### Study Limitations

Previous research has shown that vestibular exercises reveal similar DVA improvements in both patients with unilateral and bilateral vestibular hypofunction (Herdman et al., 2003; Herdman et al., 2007). Non-vestibular oculomotor mechanisms such as anticipatory slow eye movements and pre-programmed catch-up saccades with short latencies could be the reason for improvement in DVA (Vital et al., 2010). However, our study did not investigate catch-up saccade properties in relation to DVA and, therefore, it is not possible to explain the improvement in terms of more effective saccades (Vital et al., 2010). DVA was tested during horizontal head movements only. Since the participants also moved their head up and down, the vertical DVA test should also have been used to assess possible improvement in the sagittal plane.

Another limitation relates to the gait analysis. We focused mainly on gait speed during the ETGUG; however, a more important underlying variable related to gait in individuals with vestibular dysfunctions is variability of gait (Schniepp et al., 2012), where, especially, temporal parameters of gait are affected in these individuals (Ilg et al., 2007; Perring and Summers, 2007). In future trials with vestibular dysfunction, gait variability can be measured reliably (Schmidheiny et al., 2015) and, thus, more comprehensive assessment of gait should be considered. This will have a better potential to fully elucidate the relation between VR-based interventions and its effect on gait variability. Regarding the interventions, a limitation is that the participants had to constantly check their feet positioning on the pressure-sensitive game plate. Therefore, the participants had to look down regularly and, furthermore, they also constantly moved their head in accordance with the moving game projection. While this meant that the aim to facilitate head movement during game-play was implemented successfully, a limitation was that the research assistant only controlled head movements visually and no instrument quantifying head movements was used. Future trials in clinical populations should also consider longer follow up periods. From our proof-of-concept study design, we are unable to determine how long the observed beneficial effects of the exergame training will last. It cannot be excluded that the improvements are only present on the final training day, after which performance drops to the baseline again. To exclude short-lived effects, with limited therapeutic value, patients should be followed-up for longer time periods in forthcoming clinical trials.

## Conclusion

The results of this proof-of-concept study suggest that the use of exergaming that requires active stepping movements and head movements during gameplay is feasible and facilitates gaze stability during head movements in healthy community dwelling older adults. Aspects of functional gait and gait initiation improve also. Future research aiming at testing this exergaming intervention in patients suffering from vestibular impairments is warranted.

## Author Contributions

JS developed the research question. The concept and design part was established by JS, KW, and EdB. KW did data acquisition. JS, KW, and DS did the analysis and interpretation of the results. JS and KW produced an early version of the manuscript. EdB and DS revised the manuscript to bring it to its current version. All authors have read and approved the final manuscript.

## Conflict of Interest Statement

EdB was a co-founder of dividat, the spin-off company that developed the video games used in this study, and is associated to the company as an external advisor. No revenue was paid (or promised to be paid) directly to EdB or his institution over the 36 months prior to submission of the work. The remaining authors declare that the research was conducted in the absence of any commercial or financial relationships that could be construed as a potential conflict of interest.

## Supplementary Material

FIGURE S1 Game scores.

TABLE S1 Data underlying the analysis presented in the manuscript.
