# Supplementary figures and images for: Exergaming in a Moving Virtual World to Train Vestibular Functions and Gait; a Proof-of-Concept-Study With Older Adults
Source: Front Physiol. 2018 Jul 31;9:988. doi: 10.3389/fphys.2018.00988 (PMC6080593; doi:10.3389/fphys.2018.00988)

Supplementary file

Game scores

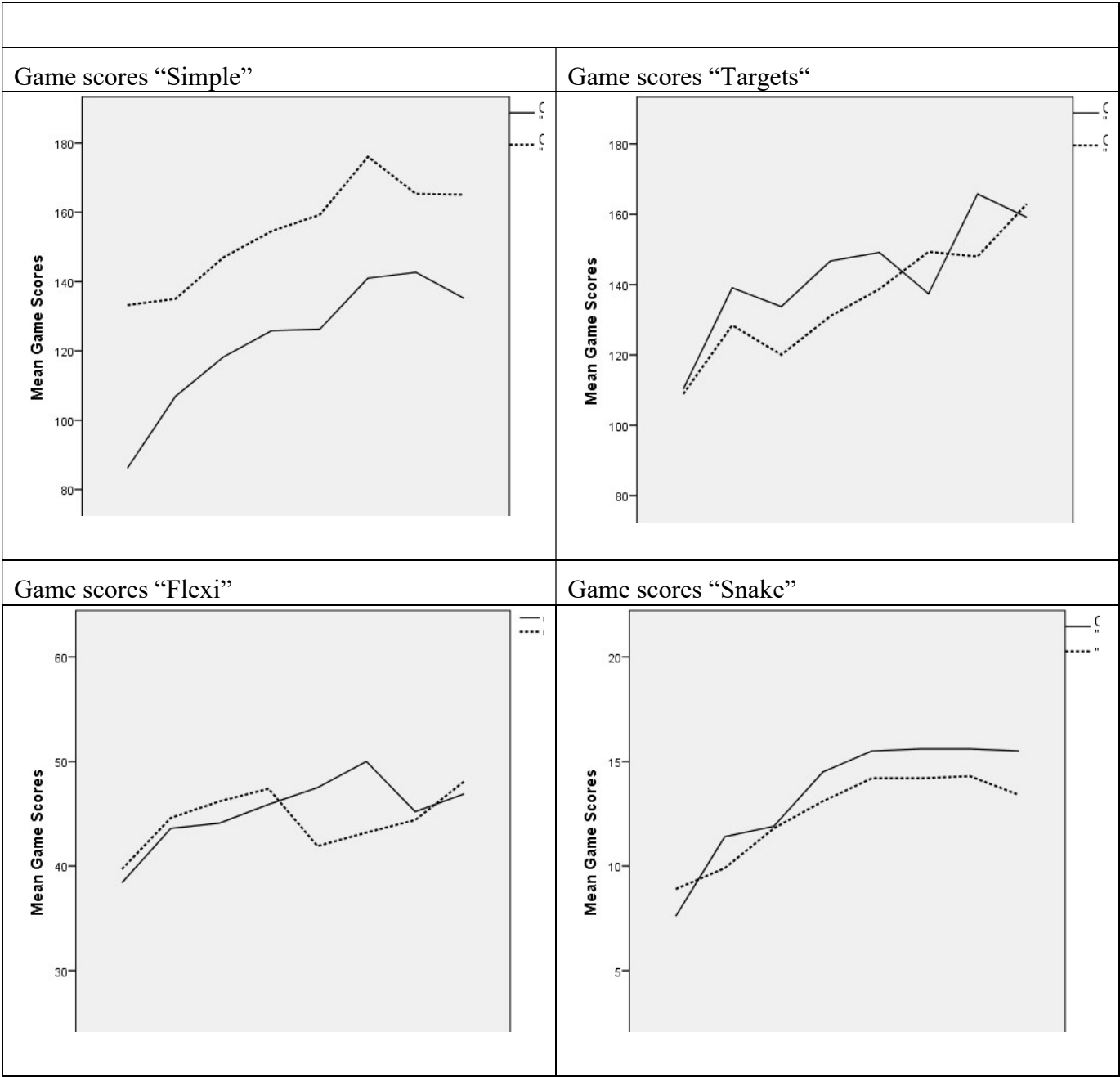

Targets (Hits and Misses)

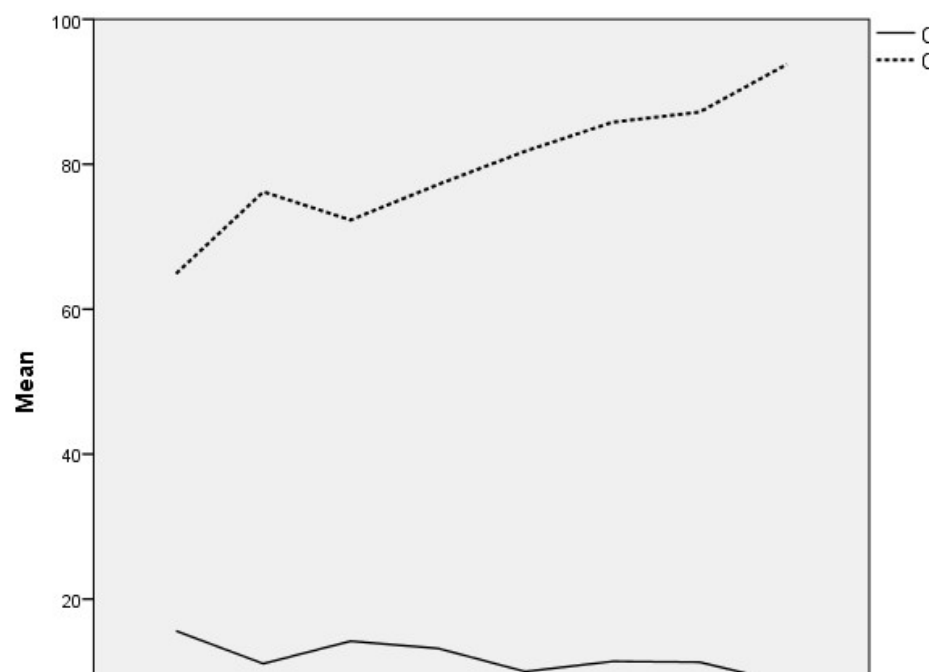

Supplement: FIGURE S1 — Game scores. [file Image_1.pdf]
